# Evidence-Based Translational Strategy of Medicated Topical Gel for Diabetic Wound Management

**DOI:** 10.3390/pharmaceutics18040429

**Published:** 2026-03-31

**Authors:** Poonam Pal, Santosh Kumar, Ankita Yadav, Salil Dubey, Sanchit Arora, Sanjay Kumar, Mayank Gangwar, Anurag Mishra, Amaresh Kumar Singh, Shreyans K. Jain, Ashish Kumar Agrawal, Satyanam Kumar Bhartiya, Sanjeev Kumar

**Affiliations:** 1 Department of Dravyaguna, Faculty of Ayurveda, Institute of Medical Sciences, Banaras Hindu University, Varanasi 221005, India; poonambhu230@gmail.com (P.P.); santoshc10494@gmail.com (S.K.); ankitayadav9454@gmail.com (A.Y.); mishraanurag.bhu@gmail.com (A.M.); 2 Department of Zoology, Institute of Sciences, Banaras Hindu University, Varanasi 221005, India; salildubey@bhu.ac.in (S.D.); amareshsingh@bhu.ac.in (A.K.S.); 3 Department of Pharmaceutical Engineering and Technology, Indian Institute of Technology, Banaras Hindu University, Varanasi 221005, India; sanchitarora.rs.phe24@itbhu.ac.in (S.A.); sanjaykumar.phe19@itbhu.ac.in (S.K.); sjain.phe@iitbhu.ac.in (S.K.J.); ashish.phe@iitbhu.ac.in (A.K.A.); 4 Clinical Studies and Trials Unit, Development Division, Indian Council of Medical Research, New Delhi 110029, India; mayank.gangwar@icmr.gov.in; 5 Department of General Surgery, Faculty of Medicine, Institute of Medical Sciences, Banaras Hindu University, Varanasi 221005, India; satyanambhartiya42@gmail.com

**Keywords:** diabetic wound healing, medicated topical gel, dermal toxicity, markers

## Abstract

**Background:** Chronic diabetic wounds represent substantial issues in healthcare due to their poor healing rate and susceptibility to hyperglycemia, infections, and other serious consequences. **Methods:** In this work, we developed a Medicated Topical Gel (MTG) that promotes healing of diabetic wounds. The MTG’s distinctive qualities, including biocompatibility, stability, affordability, cost-effectiveness, and non-toxicity, were evaluated in a dermal toxicity assessment as well as a diabetic wound assay from 0 to the 24th day of the study. **Results:** MTG treatment significantly accelerated wound closure compared with other formulations. In vivo studies revealed that diabetic wounds in the rat model healed more rapidly within the 24-day study period when treated with MTG. Western blot analysis revealed a significant decrease in pro-inflammatory markers, accompanied by enhanced angiogenesis, which was further confirmed by histopathological observations. These findings indicate that MTG effectively promotes faster wound healing by modulating inflammation and stimulating blood vessel formation. Furthermore, clinical cases have demonstrated substantial wound healing, with most cases showing significant recovery on follow-up intervals at 15th and 30th day. **Conclusions:** Our findings demonstrate a strong evidence-based therapeutic potential of MTG as an effective treatment for chronic diabetic wounds healing. They also provide a promising strategy for wound management in both the experimental and clinical case studies.

## 1. Introduction

Diabetic wound healing is a serious condition for the whole world [1]. Diabetes, a very challenging metabolic disorder that affects an estimated 537 million adults globally between the ages of 20 and 79 years, is a long-term condition that results from inadequate insulin production. Since the first edition in 2000, the projected prevalence of diabetes in adults aged 20 to 79 has more than tripled, from an estimated 151 million people (4.6%) to 537.5 million people (10.5%) of the world’s population today [2]. The male and female are equally affected by diabetes and the majority of patients have no idea how to take care of a diabetic wound. Long-term diabetic patients get wounds that do not heal. At present, diabetic wounds are a major threat for a diabetic person with a 66% of recurrence and 12% of amputations rate [3]. Particularly for wound healing, growth factors initiate the physiological mechanisms that produce angiogenesis, granulation tissue formation, and immune response mitigation. The precise cellular response is dependent on the kind of cell and the surrounding environment, and a single growth factor may be in charge of thousands of activities [4,5]. Few studies have identified important molecular targets that contribute to delayed healing, including vascular endothelial growth factors (VEGF), tumor necrosis factor-α (TNF-α), and interleukin-6 (IL-6) [6]. Diabetes can lead to either enhanced or decreased angiogenesis, depending on the pathological process. Diabetic wounds have a deficit in angiogenesis, and an inadequate blood supply restricts the delivery of necessary nutrients and oxygen, which makes wound healing challenging [7]. In the case of wound healing, endothelial cells (ECs) are involved in the local and synthetic stimuli by responding to apoptosis, proliferating, and migrating in response to cues. During vascular trauma, the vascular endothelial growth factors (VEGF) and their receptors, like the VEGFR-1/2, act mainly in ECs, providing their survival, proliferation, or migration in injury conditions [8,9]. The destruction of tissue is mediated via the release of the lytic enzyme by the inflammatory mediators/cells under the influence of TNF-α [10]. It has also been noted that Inhibition of TNF-α leads to expediting the tissue repair process and in diabetic patients, TNF-α inhibition reduces apoptosis in the bone lining [11]. An elevated level of TNF-α in the skin blister causes impaired neutrophil apoptosis. Interleukins are the key participants during tissue repair and wound healing [12]. There are multiple reports indicating the role of IL-6 in wound healing due to its pleiotropic nature. IL-6 modulates the endothelial cells by influencing their proliferation and differentiation in order to influence cutaneous wound healing [13]. It acts as a chemotactic agent at wound sites for the migration of leukocytes and also influences fibroblast migration at wound sites via JAK/STAT and MAPK signaling pathways. Being pleotropic in nature, IL-6 not only acts as a pro-inflammatory molecule, but also shows pro- and anti-fibrotic effects that make its role complex in the context of wound healing [14,15]. Impaired cutaneous wound healing is a significant pathological consequence in diabetic patients. These chronic, non-healing wounds frequently progress to serious conditions such as microbial infection, tissue necrosis, gangrene, limb loss or lower-extremity amputation, and in severe cases may lead to life-threatening complications or mortality [16]. Conventional therapies are often costly and show delayed or inadequate healing. Therefore, advanced research is needed to explore novel therapeutic strategies to deal with this emerging issue. One such approach involves the use of medicinal plants as sources of natural products for the discovery of new therapeutic agents for the management and treatment of diabetic wounds [17]. Herbal medications are now much sought after for primary healthcare in underdeveloped nations due to their greater cultural tolerance, better bodily compatibility, and fewer adverse effects. 75–80% of people worldwide utilize herbal medication for primary healthcare, primarily in underdeveloped nations [18,19]. Numerous phytochemical components found in medicinal plants are useful in preventing persistent wound infections and speeding up the healing process [20]. India possesses a rich heritage of plant-based healthcare knowledge, and numerous Ayurvedic plants play a significant role in wound healing. Numerous plants and their preparations, such as pastes, decoctions, and extracts, are widely used by tribal communities and in folk traditions in India for treating cuts, wounds, and burns [21]. Medicinal plants facilitate wound healing by naturally enhancing the body’s intrinsic repair systems [22]. The present study was carried out to evaluate the therapeutic potential of MTG in diabetic wounds. MTG was prepared using the root of *Decalepis hamiltonii* Wight & Arn, and the seed oil of *Balanites aegyptiaca* Linn. Delile as a new proprietary preparation. The selected herbs of MTG were reported to have significant antioxidant, antimicrobial, anti-inflammatory, and wound healing properties. The roots of *Decalepis hamiltonii* have been used in Ayurveda since ancient times and are known to stimulate appetite. They are traditionally employed in the management of skin diseases, hemorrhoids, rheumatism, asthma, and bronchitis, and are also recognized for their diaphoretic, antiviral, and general tonic properties [23,24]. *Decalepis hamiltonii* Wight & Arn, commonly known as swollen root (family Asclepiadaceae), is a climbing shrub found in the forests of Peninsular India and in the Southern area known as Maredu kommulu, Nannari kommulu [25]. *Decalepis hamiltonii* root Wight & Arn is used in India as a substitute for the well-known Ayurvedic medication *Hemidesmus indicus* (L) R. Br., which has medicinal characteristics [26]. Administration of *Decalepis hamiltonii* root extract to STZ-induced diabetic rats promoted antioxidant defenses, reduced oxidative stress, DNA damage, ameliorated liver injury and diabetic complications [27]. The antidiabetic and hypolipidemic potential of the ethanolic extract of *Decalepis hamiltonii* root was assessed in induced diabetic rats for 28 days at various doses. The alcoholic root extract showed significant therapeutic effects against diabetes and dyslipidaemia [28]. Research findings indicate that the root of *Decalepis hamiltonii* exhibits anti-inflammatory properties through suppression of pro-inflammatory cytokines such as TNF-α and IL-2, likely due to bioactive constituents including lupeol acetate and 5,7,4-trihydroxy flavanone-4′-*O*-β-D-glucoside [29].

*Balanites aegyptiaca* Linn. Delile is also known as desert dates. It is distributed in the arid zone of India, including western Rajasthan and peninsular areas extending from southeast Punjab to West Bengal and Sikkim. In Egyptian traditional medicine, its fruits are used for diabetes management because of their hypoglycemic properties. Additionally, *B. aegyptiaca* has been reported to possess potential wound-healing activity [30]. Various parts of *B. aegyptiaca*, such as the seeds, fruits, bark, roots, and leaves, have been widely used in traditional medicine for treating ailments like skin diseases, wounds, dysentery, malaria, jaundice, syphilis, epilepsy, and intestinal worms [30,31,32]. The presence of bioactive compounds and elements such as sulfur and silver in *B. aegyptiaca* seed oil may be responsible for its ability to enhance wound contraction and promote epithelialization in chronic diabetic wounds. *B. aegyptiaca* seed oil is cheap, chemical-free, and versatile, and it is anticipated to be a valuable therapeutic and economic remedy for wound healing [33]. GC-MS analysis of *B. aegyptiaca* seed oil extracted with ether revealed several bioactive compounds associated with wound healing. Compounds such as tetradecamethylcycloheptasiloxane promoted tissue formation and re-epithelialization, while feruloyl-caffeoyl-glycerol and decamethylcyclopentasiloxane exhibited strong antioxidant activity. Additionally, 17-octadecynoic acid and other constituents showed notable antimicrobial and antibacterial effects. Collectively, these phytoconstituents contribute to the wound-healing potential of *B. aegyptiaca* seed oil [34]. *B. aegypitiaca* seed oil has caffeic acid, which is very impactful for diabetic wound healing [35]. The increasing popularity of herbal medicines, easy availability of raw materials, cost-effectiveness, and paucity of adverse effects encouraged us to evaluate the wound-healing efficacy of MTG. Additionally, gels help the wound area retain moisture, which is essential for angiogenesis, keratinocyte migration, collagen production, and reduced scar formation for both acute and chronic wounds [36,37,38].

Therefore, in the present study, MTG was developed and evaluated through characterization and toxicity assessment, followed by its application in the treatment of diabetic wounds in a rat model using wound healing assays, histopathology, and molecular analysis, along with evidence-based clinical evaluation. The formulation is based on Beewax and SEPINEO™ P 600 gel and medicated oil using Ayurvedic methodology incorporated into the *D. hamiltonii* root powder and *B. ageyiptica* seed oil without adding any synthetic material. Its biodegradable and non-toxic components demonstrate strong potential for pharmaceutical applications. However, MTG exhibited poor water solubility, necessitating the use of a solvent for dissolution, which may compromise its efficacy. Therefore, SEPINEO™ P 600 gel was predominantly used in the topical formulation due to self-gelling, thickening, and emulsifying properties. It is a concentrated dispersion of copolymer that, upon hydration, forms a 3D network, imparting high viscosity at low concentration and maintaining stability at elevated temperature, thereby enhancing the formulation stability, emulsifying performance, and texture [39,40]. Beewax (*Apis mellifera*), a natural lipid-based substance, has been utilized in pharmaceutical drug delivery systems. However, its potential application in dermatological formulation has been relatively unexplored [41]. Beeswax is a promising candidate for developing nanosystems for topical treatments due to its ability to remain intact after incorporation into an oil/water (o/w) based cream, providing benefits similar to standard dermatological formulations. Beeswax is commonly employed as a thickening and humectant in ointments, creams, and other formulations [42].

## 2. Materials and Methods

### 2.1. Plant Material Collection and Authentication

The fruits of *Balanites aegyptiaca* were procured from Sinhal Herbs, Neemuch, Madhya Pradesh, India (GSTIN/UIN:23HNFPS4131H1Z0), while the roots of *Decalepis hamiltonii* were procured from Agasti Agroved, Pune, Maharashtra, India (GST:27ABCFA4195K1Z7). Both plant materials were authenticated by the Department of Dravyaguna, Faculty of Ayurveda, Institute of Medical Sciences, Banaras Hindu University, Varanasi, Uttar Pradesh, (221005), India. Corresponding herbarium specimens (DG/24-25/903-904) have been deposited in the Department of Dravyaguna.

### 2.2. Sample Preparation

*Decalepis hamiltonii* roots (DHR) were dried and ground into a coarse powder using a high-grade iron mortar. The powder was macerated in water at a ratio of 100 g root material to 1 L of water (1:10) for 72 h at 25 °C. This extraction procedure was performed three consecutive times. The combined extracts were subsequently lyophilized, yielding approximately 8% dried extract. Freshly collected *Balanites aegyptiaca* fruits (100 kg) were exposed to mild sunlight to remove surface impurities. After thorough drying and cleaning, the hard outer shells were cracked to retrieve the seeds. From 15.7 kg of processed fruits, about 10% seeds were recovered. The seeds were then subjected to oil extraction using an Imperium Oil Press Machine (1500 W), Imperium Ventures, Bengaluru, Karnataka, India, producing approximately 55% oil.

### 2.3. Chemical Reagents Used

SEPINEO^TM^ P 600 was a gift sample from Seppic (Paris, France), Streptozotocin (STZ) (HiMedia, Thane, Maharashtra, India), while citrate buffer pH 4.5 and 10% neutral buffer formalin were purchased from R&M Chemicals (Evergreen Engineering & Resources, Semenyih, Malaysia), Selangor, Malaysia. The glucometer and test strips were procured from ACCU-CHEK^®^, Mumbai, Maharashtra, India. DiD (DiIC18(15); 1,1′-dioctadecyl-3,3,3′3-tetramethylindodicarbocynanine, 4-chlorobenzene sulfonate salt) was procured from Thermo Fisher, Waltham, MA, USA. HPLC-grade acetonitrile, methanol, acetic acid and water were purchased from Merck, Bangalore, India.

### 2.4. Development of Medicated Topical Gel (MTG)

#### 2.4.1. Phase I—Preparation of Medicated Oil (Conditioning and Purification of Crude Oil)

Before performing *Sneha Siddhi Kalpana* (medicated oil processing), the Sneha Dravya (base oil) undergoes an essential purification procedure known as *taila murchana* (Ayurvedic pharmaceutico-processing). During the Sneha Paka (oil cooking process), the paste (kalka) and decoction using *D. hamiltonii* root powder (6.80% in water (87.76%) and *B. aegyptiaca* seed oil (5.44%) [43]. This step is intended to eliminate undesirable qualities such as *durgandha* (unpleasant odor), *ama dosha* (impurities/toxins), and *ugrata* (harshness) present in the raw oil. Through this purification, the oil attains improved fragrance, appearance, and enhanced capacity to absorb active principles during *Sneha Paka*. Moreover, it acquires additional therapeutic benefits from the *Murchana Dravyas*. For the *Balanites aegyptiaca* seed oil, 800 mL of *Ingudi* seed oil was placed in a stainless-steel container and heated mildly over *mandagni* (approximately 150–200 °C). Within a short period, a white frothy layer appeared. The heating was continued for about 30 min until the froth completely disappeared. The oil was then cooled naturally at room temperature [44].

#### 2.4.2. Phase II—Preparation of MTG

**Figure 1:**
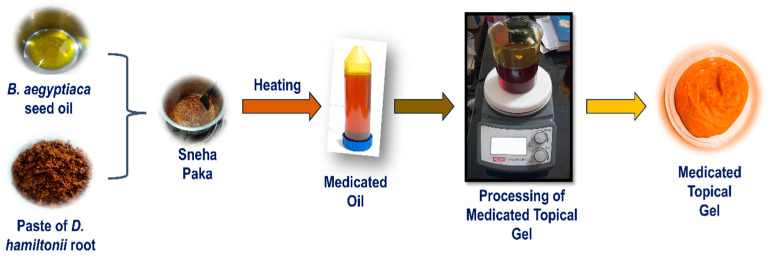
Development of Medicated Topical Gel (MTG).

MTG was formulated using *D. hamiltonii* root powder and *B. aegyptiaca* seed oil, along with SEPINEO™ P 600 and beeswax. To begin, the medicated oil phase was prepared by combining it with beeswax and heating the mixture to 50 °C. This was maintained under continuous magnetic stirring at 200 rpm for 30 min until the beeswax fully melted and a uniform oil phase formed. After slight cooling (35 °C), SEPINEO^TM^ P 600 was gradually added under continuous stirring at 200 rpm for 3 min to facilitate proper gel network formation (Table 1). The formulation was then cooled to room temperature under gentle stirring to maintain uniformity and stability (Figure 1). SEPINEO™ P 600, a multifunctional polymer, serves as a thickening, emulsifying, and stabilizing agent, contributing to the desired texture and stability of the formulation. Beeswax acts as an emulsifier and rheological modifier by promoting fine crystallite formation and preventing phase separation of the oil [39,40].

### 2.5. Physiological Characterization of MTG 2.5.1 Organoleptic Analysis

The organoleptic properties of the MTG (homogeneity, texture, color, stability, and odor) were observed.

#### 2.5.1. Microscopic Analysis

A Dewinter microscope, New Delhi, India with a photo-digital camera was used to record the microscopic image of MTG formulations after a 24 h storage period. The microscopic images taken 6 h after the MTG was created (Figure 3A) indicate that semisolid emulsions have a large number of tiny droplets with a very homogeneous particle size distribution, suggesting that the manufactured gel has excellent stability.

#### 2.5.2. Determination of pH

After dispersing one gram of the gel formulation in 100 mL of deionized water, it was kept for 2 h. An Eutech pH meter (Thermo Fisher, Singapore) was used to measure the formulations’ pH at room temperature. The pH of the gel was measured three times. The mean and +/− standard deviations were determined [45].

#### 2.5.3. Determination of Viscosity

The Brookfield viscometer DVE (AMETEK Brookfield, Mumbai, Maharashtra, India) with LV spindle number 52 was used to measure the MTG’s viscosity. The viscometer spindle was placed in a beaker with 20 g of MTG and spun at room temperature at 50 rpm. The viscosity of the gel was tested three times [46]. The mean values and the ±standard deviations were calculated.

#### 2.5.4. Spreadability

MTG’s Spreadability was determined using the parallel plate method [46] and screened using the Spreadability test. To put it simply, 0.5 g of the MTG was carefully placed into a glass plate that had previously been marked with a 3 cm diameter circle. The circle’s Spreadability was assessed as it grew after the gel dispersed. Fresh samples were used for each experiment three times.

#### 2.5.5. Surface Morphology

Surface morphological features of the MTG were examined using high-resolution field emission scanning electron microscopy (Carl Zeiss, Jena, Germany, Model: GEMINI 560). A single drop of MTG was placed on a glass slide and gently covered with a coverslip. The droplet was evenly distributed and allowed to dry overnight under vacuum. Subsequently, a thin layer of carbon was coated on the dried sample, and it was imaged using FE-SEM. The imaging procedure for the lyophilized MTG sample was performed following the same methodology.

#### 2.5.6. Powder X-Ray Diffraction (PXRD) Analysis

Powder X-ray diffraction measurement for the MTG was performed on a XRD (Bruker Model D8 Advance (Eco)), Bremen, Germany, diffractometer system.

#### 2.5.7. Differential Scanning Calorimeter (DSC) Analysis

Thermal properties were examined by the DSC curves of the MTG, which were obtained using simultaneous differential scanning calorimetry (DSC), model DSC2500, manufactured by TA Instruments, New Castle, DE, USA. MTG was performed in triplicate using approximately 5 mg of the MTG sample, alumina sample container, under a nitrogen atmosphere with a flow of 20 mL min^−1^, at a heating rate of 10 °C min^−1^ up to 500 °C.

#### 2.5.8. Thermogravimetry (TGA) Analysis

The Thermogravimetric Analyzer, Mettler-Toledo (Columbus, OH, USA) was used to measure the thermal analysis of MTG. TGA was carried out in accordance with ASTM method E1641-07 with a heating rate of 10 °C per minute, ranging up to 500 °C, and nitrogen flow (20 mL/min).

#### 2.5.9. Fourier Transform Infrared Spectroscopy (FTIR) Analysis

The MTG was scanned in the range of 400–4000 cm^−1^ under the IR region using an FT/IR-4700 spectrophotometer (JASCO Corporation, Tokyo, Japan), and associated functional groups were determined [47].

#### 2.5.10. Drug Stability Testing by the HPLC

Drug stability testing is designed to estimate the shelf life, conditions for storage, and labeling requirements while assessing the impact of external influences on the drug product’s quality. Any pharmaceutical product must undergo stability testing to ensure that its quality, safety, and potency remain intact during its shelf life. Although these studies are time-consuming and expensive, they give scientific expertise and should be planned in compliance with the standards established by the ICH, WHO, and EMA. High-Performance Liquid Chromatography is an important analytical tool for determining the stability of pharmaceutical drugs. HPLC can separate, identify, and measure the active component, as well as any impurities that may have formed during the drug product’s manufacturing and storage. Studies on forced deterioration should be carried out to discover the degradation pathways and degradation products produced during storage [48,49]. A 40 mg MTG dissolved in 2 mL of HPLC-grade methanol and sonicated for 5 min. After that, the sample was centrifuged at 12,000 RPM for 10 min. The supernatant was transferred to another fresh centrifuge tube and again centrifuged at 12,000 RPM for 5 min. The supernatant was collected and kept for 5 min to assess sample clarity. The 10 microliters of clear sample were injected into the HPLC. Chromatographic separation was performed using a reversed-phase HPLC system equipped with a C18 column (Shimadzu, 4.6 × 150 mm, 5 µm particle size). The mobile phase consisted of solvent A (water containing 0.1% acetic acid) and solvent B (acetonitrile:methanol, 50:50 *v*/*v*). The mobile phase was delivered at a total flow rate of 0.75 mL/min, with solvent A and solvent B at 0.20 mL/min and 0.55 mL/min, respectively. The total run time for the analysis was 30 min.

### 2.6. In Vivo Analysis

#### 2.6.1. Animal Model

Non-pregnant and nulliparous female Charles Foster rats (200 ± 50 g weight) were procured from the Institute’s animal facility. All the animals were maintained under standard laboratory conditions, including a temperature of 23 ± 3 °C, relative humidity of 50–60%, and 12 h light/dark cycle. The animal was maintained on a standard rat pellet diet with free access to water (ad libitum). All experimental protocols were approved by the Institutional Animal Ethics Committee (Dean/2024/IAEC/6885) and were conducted in compliance with the CCSEA guidelines.

#### 2.6.2. Experimental Design for Acute Toxicity and Repeated Toxicity

Dermal toxicity was assessed in acute and repeated exposure. To conduct acute toxicity, OECD guidelines (402) were adopted with some modifications [50]. Whereas a repeated toxicity experiment was performed by following OECD guidelines (410) with some modifications [51]. For acute (14 days) dosing, animals were divided into four groups: each group had three animals (*n* = 3): control, DHRE, BASO, and MTG, whereas repeated (28 days) dosing involved R-control, R-DHRE, R-BASO, and R-MTG groups with three animals in each group. To test the effect of the drug, a single dose of 2000 mg/kg B.W. was administered for 14 days (acute) and repeated doses for 28 days (repeated dosing) were applied. Animal kept under observation for epidermal and behavioral changes. After completion of respective time points, rats were narcotized by the intraperitoneal administration of ketamine and xylazine (80:20) mg/kg bodyweight. Blood samples were collected through the retro-orbital vein, and animals were humanely sacrificed using a high dose of anesthesia and a drug applied to the dorsal region. Skin tissue from all groups was collected for histology.

#### 2.6.3. Biochemical Examination

Post completion of the dermal application after 14 and 28th days, blood samples from all the groups were collected in non-coated tubes for biochemical parameter analysis. Biochemical parameters (Table 3) were analyzed by using an automatic blood cell analyzer (Arkray’s auto hematology analyzer, Kyoto, Japan) [52,53] using serum samples.

#### 2.6.4. Diabetic Induction

In this study, after an overnight fast, diabetes was induced in the experimental groups by administering a single intraperitoneal (IP) injection of streptozotocin (STZ) at a dose of 45 mg/kg body weight. Each rat was prepared according to its body weight after STZ was newly dissolved in citrate buffer (pH 4.5) and shielded from light.

The injection was carried out using an insulin syringe. Instead of providing 10% sucrose water for drinking for 6 h after STZ injection, to prevent fatal hypoglycemia. Blood glucose levels were measured 72 h post-injection via tail vein sampling using an ACCU-CHEK^®^, Mumbai, Maharashtra, India, glucometer. Streptozotocin (STZ), originally identified in 1959 as a natural antibiotic produced by *Streptomyces achromogenes*, is well known for its selective toxicity to pancreatic β-cells. The STZ molecule (molecular formula: C_8_H_15_N_3_O_7_; molecular weight: approximately 265 g/mol) comprises two functional components: a glucopyranosyl group, which facilitates its uptake into pancreatic β-cells through the glucose transporter GLUT2, and a nitrosourea moiety, which is responsible for β-cell destruction [54].

#### 2.6.5. Excisional Wound Model

Streptozotocin (STZ)-induced diabetes was used to select rats with consistently high blood glucose levels. Described a full-thickness excisional wound model. Diabetic rats were sedated with an intraperitoneal (IP) injection of ketamine and xylazine (80:20 mg/kg), followed by a 2 × 2 cm^2^ excisional wound on the thoracolumbar area of the back. The wounds remained naked and unprotected. After recovering from anesthesia, the animals were placed individually in properly disinfected cages. After creating the wound, the animal was kept in separate cages for the treatment [55].

The experimental group

- The wounds of the normal (without diabetic) rat were left untreated;
- The wound of the diabetic rat was left untreated;
- The wound of the diabetic rat treated with the marketed application of povidone–iodine 5%;
- The wound of the diabetic rat treated with the DHRE;
- The wound of the diabetic rat treated with the BASO;
- The wound of the diabetic rat treated with MTG.

#### 2.6.6. Diabetic Wound Healing Study

Animals were randomly assigned to six groups (*n* = 3), group 1: control group (no diabetics), group 2: negative control, group 3: positive control (marketed formulation povidone–iodine 5%), group 4: DHRE, group 5: BASO, and group 6: MTG. The therapeutic efficacy of the gel formulations (MTG) in boosting diabetic wound healing was evaluated using a vernier caliper to measure wound area reduction on various days until re-epithelialization was complete. Following the establishment of a diabetic wound, every drug (500 mg) was topically applied twice daily to each group, and wound healing was examined.

#### 2.6.7. Histological Examinations of Diabetic Wound Healings

Collected diabetic wound tissue (skin) samples from each animal were fixed in a 4% paraformaldehyde (PFA) solution at 4 °C overnight, and further dehydration and paraffin-embedded blocks were prepared. Tissue blocks were subjected to a 5 µm thin section (Rm2245 Leica microtome, Leica Biosystems, Nussloch, Germany) for the staining procedure. Tissue sections were stained with hematoxylin and eosin, then viewed using a bright-field microscope (Nikon Eclipse E200, Nikon Corporation, Tokyo, Japan) [52].

#### 2.6.8. Western Blot Analysis of Inflammatory and Angiogenic Marker Proteins

Western blotting was performed to evaluate the expression of key proteins involved in inflammation and angiogenesis, including vascular endothelial growth factor receptor 2 (VEGFR-2), interleukin-6 (IL-6), and tumor necrosis factor-alpha (TNF-α) [56] using β-actin as a loading control. Briefly, wound tissue samples from the untreated and treated groups were dissected on ice and immediately homogenized (~50 mg tissue) in 1 mL of ice-cold RIPA lysis buffer (20 mM Tris-HCL, pH 7.4; 150 mM NaCl; 1% Triton-X-100; 1% sodium deoxycholate; 0.1% SDS, and 1 mM EDTA) supplemented with 10 μL protease inhibitors. Homogenization was performed using a mechanical tissue homogenizer, and lysates were centrifuged at 12,000 rpm for 20 min at 4 °C to collect the supernatant. The total protein concentration was quantified using a Bradford assay. Equal amounts of protein (~20 μg per sample) were mixed with Laemmli sample buffer, boiled for 5 min at 95 °C, and resolved by electrophoresis on SDS-polyacrylamide gel. Proteins were subsequently transferred onto a PVDF membrane using a Turbo Trans-Blot semi-dry transfer system, Bio-Rad, Hercules, CA, USA. Following transfer, membranes were blocked for 1.5 h at room temperature with 5% bovine serum albumin (BSA) prepared in Tris-buffered saline containing 0.1% Tween-20 (TBST) to prevent nonspecific binding [57]. The bots were then incubated overnight at 4 °C with specific primary antibody: VEGFR-2 (D5B1) Rabbit mAb (#9698; Cell Signaling Technology 1:1000), IL 6 Rabbit pAb (#A0286; Abclonal; 1:1000, TNF-α Rabbit pAb (#A11534; Abclonal; 1:1000), and β-Actin Rabbit mAB (AC026; Abclonal; 1:1000). After washing with TBST, the membranes were incubated for 1 h at room temperature with HRP-conjugated Goat Anti-Rabbit IgG secondary antibody (ab6721; Abcam, Cambridge, UK; 1:5000). Protein bands were visualized using an enhanced chemiluminescence (ECL) substrate (Bio-Rad) and imaged using a Chemidoc Imaging System, Bio-Rad, USA. The relative intensity of the bands was quantified using ImageJ software (1.54g; Java 1.8.0_345), normalizing each target protein to β-actin. Further data was analyzed in GraphPad Prism 5.0 and statistical significance was determined by one-way ANOVA followed by Tukey’s multiple comparison test.

#### 2.6.9. Gel Occlusion and Bioimaging Study

Gel occlusion and bioimaging study of the wound region was made possible by the free DiD dye and blank gel containing free DiD dye. The Photon Imager Optima System from the Biospace Lab (Nesles-la-Vallée, France) was used to take the fluorescence images. Fluorescence signals were collected at excitation and emission wavelengths of 620 and 710 nm, respectively, at 0, 0.5, 1, 2, 4, and 6 h follofwing the administration of both free DiD and DiD-loaded gel. In the in vivo bioimaging study of wound-healing rats (*n* = 3 per group) were utilized in this study. The animals were put to sleep with a continuous infusion of 3% isoflurane. The Biospace Lab imaging software (M3Vision), Software version 3.6.1.1533, was used to determine the radiant efficiency (measured as fluorescence intensity/area/time), and the wound area was circled using the region of interest (ROI) tool.

### 2.7. Case Series and Study Center

Evidence-based practice emphasizes that clinical decision-making should be guided by high-quality research evidence. It involves the critical appraisal of the best available clinical research and the application of a hierarchy of evidence throughout the evaluation process. The present investigation aimed to characterize the clinical features and results of patients with wounds related to type 2 diabetic mellitus (T2DM).

A prospective case series was conducted at a tertiary care center. The study was conducted at the Department of General Surgery, Institute of Medical Sciences, Banaras Hindu University (BHU), Varanasi, Uttar Pradesh, India (221005), of the Sir Sunder Lal Hospital. We have enrolled five (05) patients with T2DM-associated wounds, who were enrolled and managed between November 2024 and May 2025. Patient care in this case series was guided by standardized wound management protocols. Routine surgical instruments, including sterile gloves, forceps, scissors, cotton, and normal saline, were used for wound cleaning and dressing. The amount of MTG per application is calculated by the wound size [58]. MTG was applied evenly over the entire wound bed twice daily, with the dosage adjusted according to wound size on the 0th, 15th, and 30th day. Wound sizes at baseline ranged from 4 cm^2^ to 36 cm^2^. The wound was photographed by Samsung Galaxy S20 FE, model number SM-G780F/DSM, Noida, Uttar Pradesh, India, so that it could be compared to its initial state. Pus culture and sensitivity (C/S) testing were performed at baseline (0th day) and at the end of treatment (30th day) to assess microbial status. This case series was registered with the Clinical Trials Registry of India (CTRI/2025/04/083880). Written informed consent was obtained from all participants before their enrolment in the study, in the presence of an attendant.

## 3. Results

### 3.1. Physiological Characterization of MTG

#### 3.1.1. Organoleptic Characteristics

The Medicated Topical Gel (MTG) was found to be homogeneous, effortless spreading, yellow in color, mild, and pleasant odor.

#### 3.1.2. pH, Viscosity, Spreadability, and Drug Stability Testing by the HPLC

**Figure 2:**
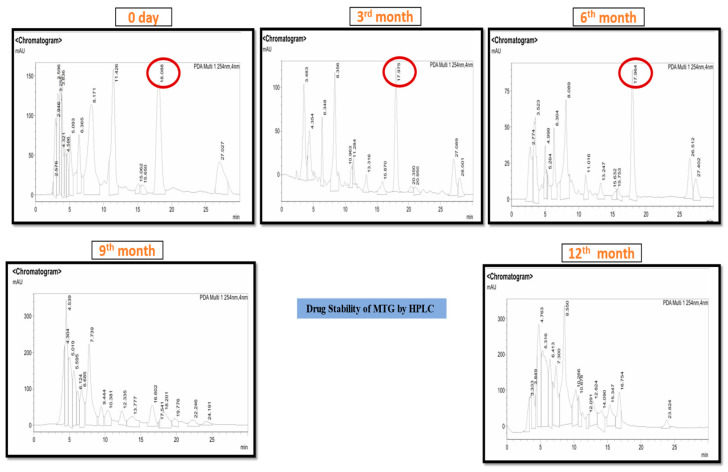
Graphical representation of MTG stability by HPLC on different time periods: 0-day, 3rd month, 6th month, 9th month, and 12th month.

The study was conducted to determine the optimal pH of the MTG at room temperature. The pH was found to be 6.8–7.0, and the viscosity was 849.6 ± 0.34 P and effortless Spreadability. The HPLC analysis confirmed the presence of three biomarkers in the MTG: Caffeic acid (RT 6.32 min), Diosgenin (RT 8.12 min), and Linoleic acid (RT 26.92 min) as identified by comparing their retention times with those of reference standards available in our laboratory (Figure S1, Supporting Information). The MTG was also subjected to long-term stability testing (general case study) under standard conditions (25 ± 2 °C/60% RH ± 5% RH) for 12 months. The stability study was performed to observe the deterioration of the present biomarkers over time 0, 3, 6 months. The chromatographic results indicated that these biomarkers remained intact in the MTG throughout the six-month study period, demonstrating the stability of the formulation. The gel remained stable for up to 6 months; however, beyond 6 months, a decline in the stability of the gel ingredients was observed (Figure 2).

#### 3.1.3. Surface Morphology

**Figure 3:**
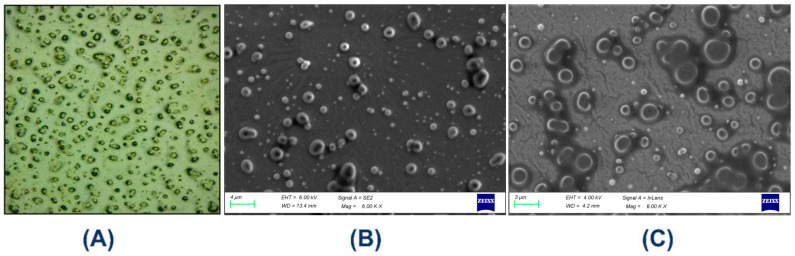
Morphological features of MTG Development of Medicated Topical Gel (MTG) (**A**) Light microscopy image (4×), (**B**) FE-SEM (6.00k×), and (**C**) FE-SEM (8.00k×).

The MTG sample’s surface morphology was examined using field emission-scanning electron microscopy (Carl Zeiss, Jena, Germany, Model: GEMINI 560). Based on SEM microphotographs taken at 6000× magnification (Figure 3B) and 8000× magnification (Figure 3C), respectively, it can be noticed that the external surface of the sample shows a dispersed gel matrix containing numerous spherical domains and homogeneity ranging from sub-micron to several micrometers, as if it were made up of spherical-shaped cluster-like structures. The observed polydispersity indicated good dispersion but potential for coalescence. Further droplet size analysis and accelerated stability testing are advised to confirm long-term physical stability.

#### 3.1.4. Powder X-Ray Diffraction (PXRD) Analysis

**Figure 4:**
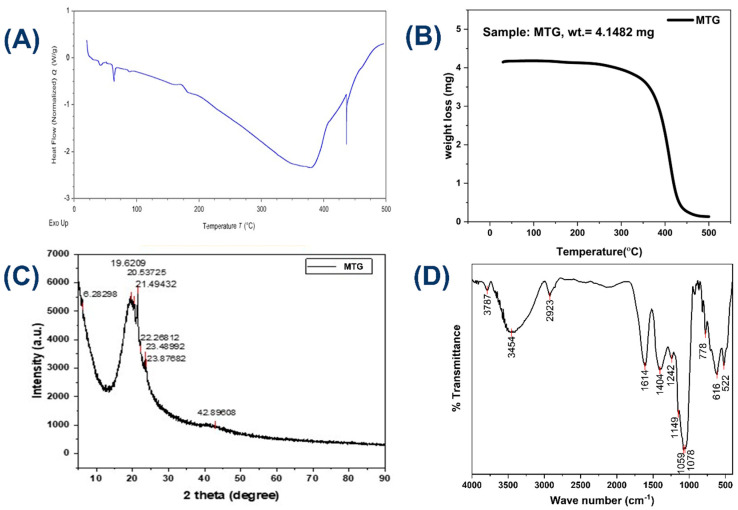
Graphical representation of MTG (**A**) DSC, (**B**) TGA, (**C**) Powder-XRD, (**D**) FTIR.

To investigate the crystallographic analysis of the as-prepared sample was performed using PXRD (Figure 4C), showing the PXRD patterns of the MTG. XRD patterns show crystalline peaks with an amorphous background, typical of botanical formulations. It can be seen that MTG has a more crystalline nature. Multiple peaks in the 19–24° range can be seen in the developed MTG, which are typical of organic crystalline compounds normally found in herbal extracts [59]. The broad peak width suggests that the plant material was mechanically processed. Based on the literature [60], peaks below 19° confirm the presence of cellulose. XRD analysis confirms the successful integration of both botanical components.

#### 3.1.5. Differential Scanning Calorimeter (DSC) Analysis

In this study, we profiled the thermal transitions of a synthesized MTG using DSC. In the thermogram (Figure 4A), it is evident that there are no significant transitions, and a minimum amount of free moisture is present. The sample shows a progressive downward endothermic shift (100–400 °C), which may be caused by water loss (evaporation), the melting of lipid constituents, or the denaturation or degradation of some biopolymers present. After 400 °C, a sharp exothermic nature is observed, and this is widely interpreted as the decomposition or combustion of organic matter. Decomposition of natural product formulations occurs at 400–500 °C due to the breakdown of residual carbohydrates, proteins, and fats [61].

#### 3.1.6. Thermogravimetry (TGA) Analysis

The TGA thermograms [61] of MTG samples show higher thermal stability (Figure 4B). MTG has a higher thermal stability. At temperatures up to 180 °C, MTG shows a very small weight loss of 1 wt.%. A small percentage of weight is lost due to moisture. Furthermore, the MTG sample exhibits a single-step decomposition process, occurring at temperatures up to 438 °C. Overall, TGA measurements showed good agreement with DSC measurements.

#### 3.1.7. Fourier Transform Infrared Spectroscopy (FTIR) Analysis

FTIR is a highly analytical method utilized to determine chemical bonds and functional groups within a given sample through the examination of its absorption patterns of infrared radiation [62]. IR spectroscopy provides information concerning the interactions of the functional groups of MTG. Fourier transform infrared (FTIR) spectroscopy is an effective technique for identifying the presence of non-covalent interactions in the self-assembled state (Figure 4D). Recordings of the spectrum of MTG exhibit peaks that are summarized in Table 2. A strong broad band depicted at 3787 and 3454 cm^−1^ is attributed to the O-H stretching vibration, indicating free and hydrogen-bonded hydroxyl groups commonly originating from the phenolic or alcoholic constituents in the plant excipients [63]. The band at 2923 cm^−1^ is ascribed to the C-H stretching of aliphatic methylene groups, which consist of the fatty acid present in natural oil or oil-based gel. The band at 1614 cm^−1^ is assigned to the C=C stretching vibrations and can be attributed to aromatic ring or conjugated alkenes, and band 1404 cm^−1^ is associated with the O-H bending, which suggests the presence of flavonoid or phenolic compounds. The band observed around 1242 cm^−1^ can be C-N stretching (amide/alkaloid) depending upon the sample, and some intense peaks at 1078 cm^−1^ and 1059 cm^−1^ are characteristic of C-O stretching vibration and typical of alcohol, polysaccharides, and ether groups, which are commonly found in the plant material-derived excipients. Another band was observed at 778 cm^−1^, which is assigned to aromatic C-H bending or ring deformed modes. And low frequency absorption at 616 and 522 cm^−1^, which falls in the far-infrared region and is likely to be associated with the C=O (amide) and C-O-C or metal oxygen associated modes respectively. These low-energy bands may indicate interaction between organic and inorganic constituents. The presence of O-H, C-H, C=C, C-N, C=O, and C-O-C bonds points to the presence of hydroxyl group (alcoholic), aliphatic methylene groups, fatty acids, aromatic ring, amide/alkaloid, carboxyl or ether groups (alcohol, polysaccharide, and ether groups), which are typical of secondary plant metabolites (Table 2).

### 3.2. In Vivo Analysis

#### 3.2.1. Biochemical Analysis of Dermal Toxicity

Biochemical results shown in Table 3A,B, compared to control, treatment groups of acute (DHRE, BASO, MTG) and repeated (R-DHRE, R-BASO, R-MTG) did not show any significant change in either hematological or biochemical parameters of acute or repeated dosing in comparison to control.

#### 3.2.2. Histological Analysis of Dermal Toxicity

**Figure 5:**
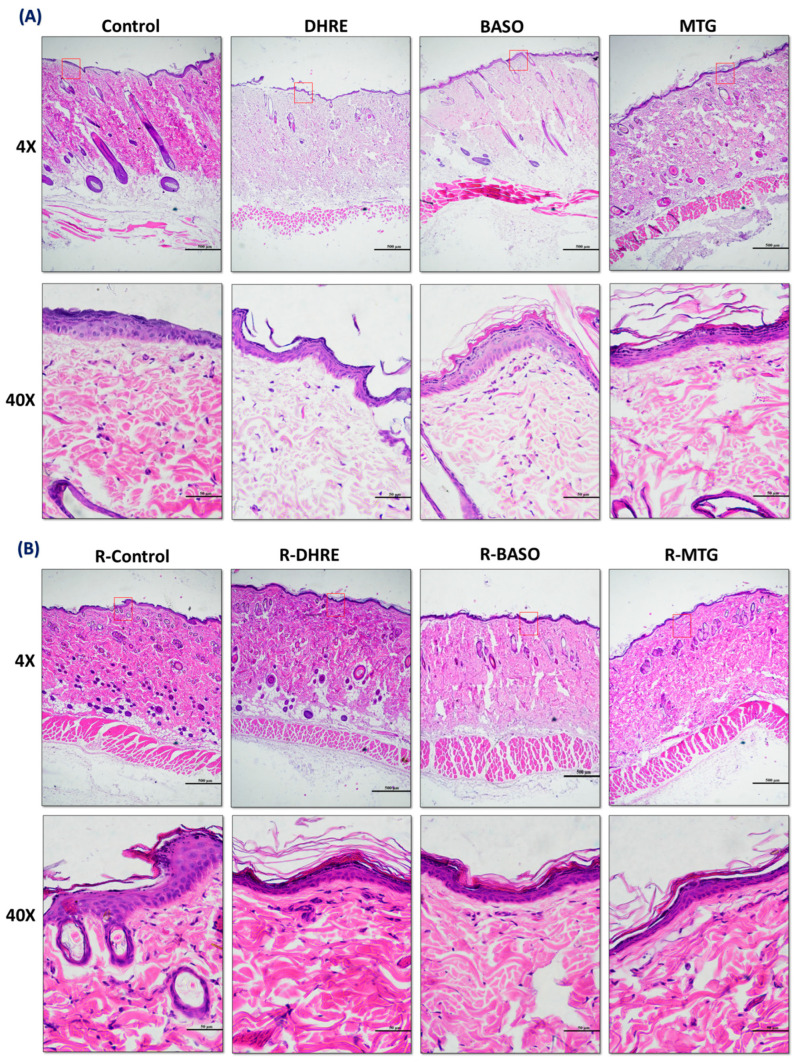
(**A**) H&E-stained LS section of all groups after single acute dosing. Images were taken in 4× (500 μm) and 40× (scale 50 μm). Single acute dosing at 2000 mg/kg for 14 days does not show any changes in epidermal region. (**B**) H&E-stained LS section of all groups after repeated dosing. The images were taken in 4× (500 μm) and 40× (scale 50 μm). In repeated dosing, R-DHRE, R-BASO and R-MTG show minute observational changes that are common in dermal and subcutaneous areas upon long term exposure to any drug. However, no changes were observed in epidermal, sebaceous or follicular areas.

The results of acute dermal toxicity (Figure 5A) show common histological features. DHRE showed thinning in the stratum spinosum layers. In the MTG group, there is a change in the shape of the cells of the stratum spinosum, and a slight atrophy in the new follicle has been observed. The result of repeated dermal and subcutaneous toxicity (Figure 5B) did not show any change in the thickness of the stratum corneum in any treatment groups compared to the control. R-DHRE showed only decreased thickness in the stratum spinosum. R-BASO groups showed a decreased number of anagen and telogen hairs in the dermal region and atrophy of mature follicles. R-MTG showed a slight decrease in thickness in the stratum spinosum and atrophy of mature follicles in the dermal region. However, no epidermal, sebaceous or follicular hyperplasia, no fibrosis, inflammation, adipocyte apoptosis, and vascular damage were observed in the hypodermis, and no difference in histoarchitecture of vein, dermal layer, stratum fibrosum and panniculus muscles layer was observed in any group.

#### 3.2.3. Wound Healing Potential Study

**Figure 6:**
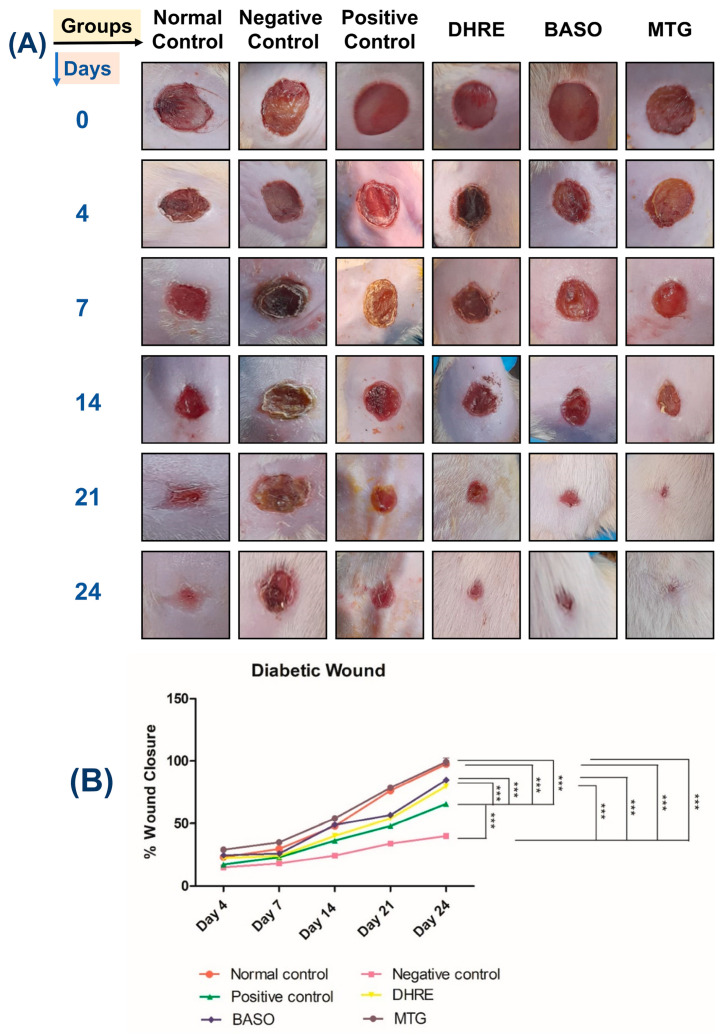
(**A**) Representative image of wounds of the normal control, negative control, positive control, DHRE, BASO, and treated group by MTG on 4, 7, 14, 21, and 24th days. Results show treatment with MTG enhanced wound healing in diabetic rats as compared to the other diabetic rat treatments. (**B**) Graphical data representation of the % wound closure rate. Error bar represents standard deviation (*n* = 3) where *** significant difference at *p* < 0.05.

The diabetic wound healing ability of MTG was tested over 24 days, with observations recorded on days 0, 4, 7, 14, and 24. Rats with STZ-induced diabetes developed excisional wounds. MTG and other compositions were given topically to the wound site. Group 1 served as the non-diabetic control, whereas the remaining groups were STZ-induced diabetic animals. It was observed that the untreated diabetic rats displayed poor or partial healing (Figure 6A). In contrast, the MTG-treated rats obtained complete wound closure by day 24. The other treatment groups—including DHRE, BASO, and the positive control—showed slower healing compared with the MTG-treated group, which was further corroborated by histological findings. The MTG-treated group demonstrated the fastest wound healing rate, reaching 99.57% closure by day 24 (Figure 6B). Among the other formulations, DHRE showed 80 ± 2.58% healing, BASO showed 83 ± 1.07%, and the positive control group showed 76 ± 0.37% healing at the end of the study. These findings clearly indicate that the MTG-treated group exhibited significantly superior wound-healing efficacy compared to all other groups. As expected, the negative control group displayed delayed healing. 

#### 3.2.4. Histopathology Analysis of Diabetic Wound

**Figure 7:**
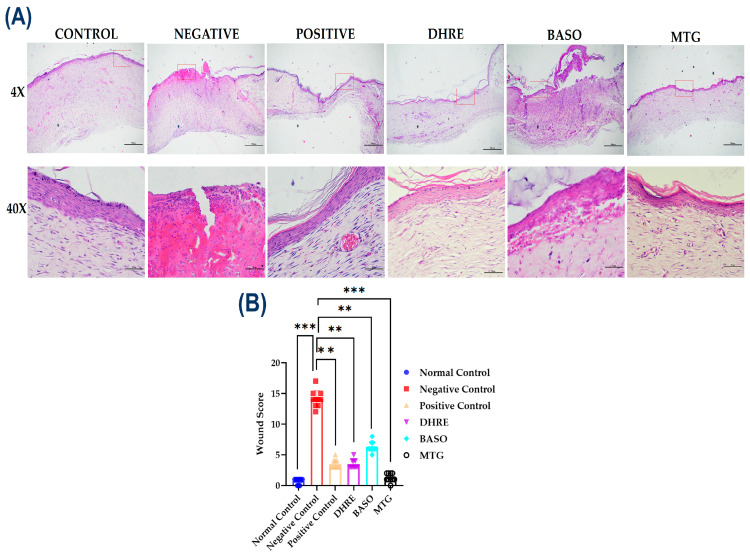
(**A**) Showing H&E stain of all experimental groups in 4× (scale 500 μm) and 40× (Scale 50 μm). (**B**) Graph showing the wound score after the end point (24 days) study of all the experimental groups. Data were analyzed in GraphPad prism 5.0 and expressed as mean ± SD (*n* = 10). Statistical significance was determined by one-way ANOVA followed by Tukey’s multiple comparison test: ** *p* < 001; *** *p* < 0.001. Graph showing the higher the wound scores the lesser the healing frequency. The MTG group has significantly lower wound score compared to negative control.

We have assessed the wound healing capacity of MTG. Criteria selected to observe the wound healing progress were edema, granulation, fibroblast, collagen, epithelialization, hyperemia, and hair follicle atrophy (Supplementary Materials). Scoring criteria designed to get a lower wound score. As depicted in (Figure 7A) and quantified in (Figure 7B), the MTG-treated group exhibited a significant reduction in wound score in comparison to the negative control (*** *p* < 0.001). BASO, DHRE, and positive control also showed mild healing (** *p* < 0.01), but among all, MTG showed the best improvement score.

#### 3.2.5. Western Blotting of Diabetic Wound

**Figure 8:**
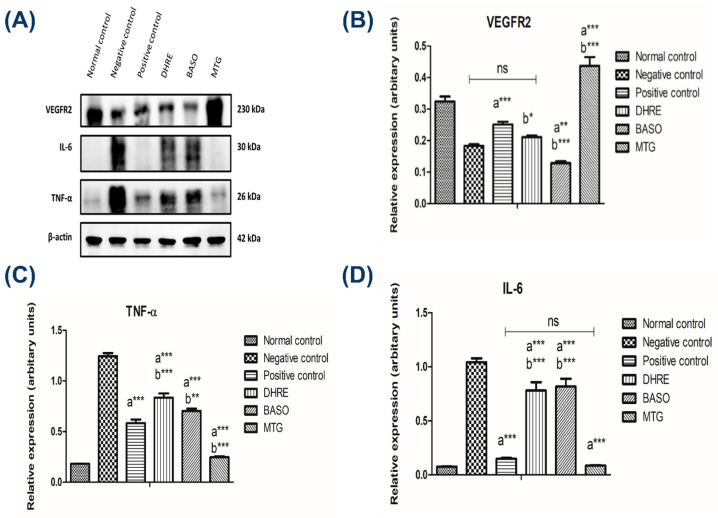
(**A**) Western blot images showing the expression levels of VEGFR, IL-6, TNF-α, and β-actin in different experimental groups: normal control, negative control, positive control, DHRE, BASO, and MTG. β-actin was used as a loading control. (**B**–**D**) Quantitative analysis of relative protein expression levels of (**B**) VEGFR2, (**C**) TNF-α, and (**D**) IL-6 in wound tissue lysates. Data are expressed as mean ± SD (*n* = 3). Statistical significance was determined by one-way ANOVA followed by Tukey’s multiple comparison test: a—compared with negative control, b—compared with positive control, ns—non-significant. (* *p* < 0.05; ** *p* < 001; *** *p* < 0.001). The MTG group exhibited significant upregulation of VEGFR2 and marked downregulation of TNF-α and IL-6 compared to the negative control and positive control, indicating enhanced angiogenesis and reduced inflammation during the wound healing process.

To understand more about the molecular mechanism contributing to the therapeutic efficacy of the test formulations, the expression of key angiogenic and inflammatory marker proteins-VEGFR-2, IL-6, and TNF-α was analyzed by Western blotting (Figure 8A). As depicted in (Figure 8A) and quantified in (Figure 8B), the MTG-treated group exhibited a significant upregulation of VEGFR-2 expression compared to the negative control group (*** *p* < 0.001). This indicates enhanced angiogenic activity, contributing to granulation tissue formation and improved oxygenation during wound repair. Moreover, VEGFR-2 expression in the MTG group was also significantly higher than that of the positive control (* *p* < 0.001). In contrast, the DHRE and BASO groups showed moderate elevation (ns) and (** *p* < 0.01) in VEGFR-2 levels relative to the negative control, whereas the negative control itself displayed minimal expression, indicating impaired angiogenic signaling. To assess the anti-inflammatory potential of the formulations, the expressions of TNF-α and IL-6 were examined (Figure 8C,D) [64]. Both cytokines were found to be significantly downregulated in the MTG-treated group compared to the negative control (*** *p* < 0.001). Relative to the positive control, IL-6 expressions were comparable and non-significant, while TNF-α expression showed significant results (* *p* < 0.001). Enhanced expression of VEGFR2 in treated samples indicated promoted angiogenesis, while reduced TNF-α and IL-6 levels reflected the anti-inflammatory potential of the treatment. Collectively, these findings demonstrated that MTG significantly (*** *p* < 0.001) enhances VEGFR2-mediated angiogenic signaling against negative control, while concurrently suppressing inflammatory cytokines TNF-α and IL-6 (*** *p* < 0.001 vs. negative control), establishing a molecular basis for its potent wound healing efficacy.

#### 3.2.6. Gel Occlusion and Bio-Imaging Study

**Figure 9:**
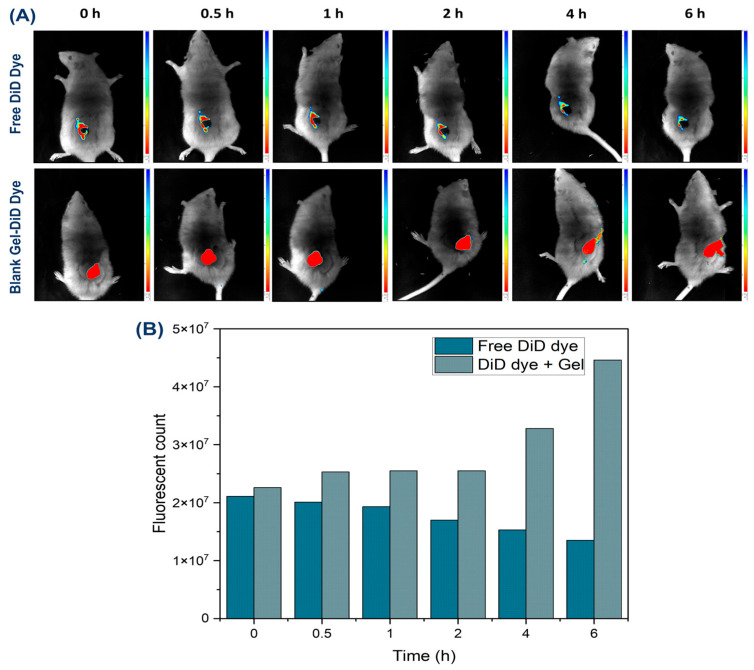
In vivo live optical imaging: (**A**) Fluorescent in vivo fluorescence imaging and gel occlusion study for the following application of the free DiD dye and blank Gel-DiD dye on the diabetic wounds, (**B**) Graphical representation of fluorescence intensity of free DiD and blank Gel-DiD dye after application to the wound at different time intervals.

In the current study, using the fluorescent DiD dye as a model marker for imaging, the IVIS study demonstrated the ability to indirectly quantify the rate and pattern of gel distribution over time. DiD dye-loaded gels were evaluated for sustained fluorescence signals that correlated with prolonged gel retention and continuous action at the rat wound site (Figure 9A). Fluorescent imaging revealed that, when monitored for up to six hours, the DiD-loaded blank gel remained localized at the application site without spreading to other body organs, confirming its site-specific behavior. Additionally, the DiD-loaded blank gel was easy to apply and maintained stable fluorescence intensity at the wound area. Fluorescence release was measured at 0, 0.5, 1, 2, 4, and 6 h post-application. The DiD fluorescence intensity was expressed as radiant efficiency (photons/s/cm^2^/sr). In contrast to the free DiD dye, which showed a rapid decline in fluorescence after administration, the DiD-loaded gel exhibited prolonged retention and a sustained fluorescent signal. This slow and steady release pattern indicates controlled dye distribution at the wound site over an extended period. The formation of a thin gel layer on the skin after application contributes to this prolonged retention. Overall, these findings support the long-term, sustained release behavior of the gel at diabetic wound sites, contributing to its potential therapeutic effectiveness (Figure 9B).

### 3.3. Case Studies

#### 3.3.1. Case 1

**Figure 10:**
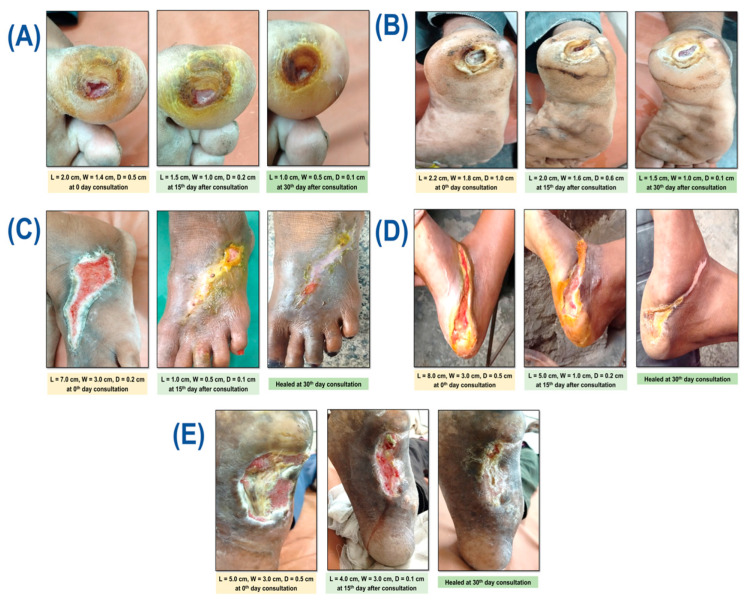
(**A**) Case 1: 46-year-old male diabetic patient, (**B**) Case 2: 62-year-old male diabetic patient, (**C**) Case 3: 52-year-old female diabetic patient, (**D**) Case 4: 48-year-old female diabetic patient and (**E**) Case 5: 54-year-old male diabetic patient treated with MTG for the wound status.

Our first patient was a 46-year-old male whose medical history included a single, inflammatory, slightly undermined, oval wound on the plantar surface of his right great toe that was about 2 cm long, 1.4 cm wide, and 0.5 cm deep at 0 days (Figure 10A). Eight years ago, he was diagnosed with type 2 diabetes mellitus. He visited the hospital with MRD number 6877209 on 1 May 2025, while receiving inconsistent medical care. The patient’s general examination showed that he was in good health, with a temperature of 98.8 °F, a pulse of 86 beats per minute, and a blood pressure of 130/88 mm Hg. Additional biochemical markers include glycated hemoglobin (HbAlc 7.2%), Complete blood count, Renal Function Test, Liver Function Test, and lipid profiles within normal ranges. A single oval-shaped wound with an irregular, callused edge and a slightly undermined wound bed with red granulation tissue and little slough was found upon local examination. There was no obvious necrotic tissue or substantial exudate. According to the patient’s medical history, he was taking broad-spectrum antibiotics and using a povidone–iodine dressing and local steroidal ointment following wound debridement. The patient, a farmer, had a history of wearing shoes that were too small or too tight. At first, the patient noticed a tiny blister that grew in size and depth over time. It was originally harmless and disregarded. The patient did not recollect any prior trauma, bug bite, or physical harm. He has no prior addiction history, chose elective treatment for a similar problem without cautious intervention; he has been on medicine since the injury, and has not made any progress, and then arrived at the hospital’s surgical outpatient department, MTG was applied following a thorough assessment of the lesion and the patient’s agreement. Blood sugar levels ranged from 140 to 160 mg/dL during fasting to 220 mg/dL after meals. Patients were instructed to apply the proper dressing after applying MTG to the wound twice a day. Additionally, the modern doctor recommended that diet and diabetes medication be continued at the same dosage. The wound was measured using a measuring scale on the 0th, 15th, and 30th day and photographed at a time point to compare it with the initial state. Furthermore, a pus sample was sent for culture and sensitivity (C/S) testing on the 0th and 30th day to determine the growth and evaluate the inhibition of bacteria in the wound following treatment, i.e., to analyze the ointment’s extra antibacterial action.

#### 3.3.2. Case 2

Our second case is a 62-year-old male who had a single undermined oval wound on the central plantar surface of his right foot for a year. The wound was inflamed with necroinflammatory debris and purulent exudate, measuring about 2.2 cm in length, 1.8 cm in breadth, and 1.0 cm in depth (Figure 10B). Ten years ago, he received a type 2 diabetes mellitus diagnosis. He was receiving inconsistent follow-up care at the same hospital on 14 May 2025, with MRD number 6994028. The patient’s general examination showed that he was in good health, with a temperature of 100 °F, a pulse of 92 beats per minute, and a blood pressure of 130/74 mm Hg. Other biochemical markers, including glycated hemoglobin (HbAlc 8.4%), Complete blood count, Renal Function Test, Liver Function Test, and lipid profiles, were within normal ranges. Upon local examination, the wound was found to be a single circular wound with uneven edges that was undermined and had a visible cavity with slough accumulation. and encircled by tissue that is hyperkeratotic. There was a considerable purulent flow, necrotic tissue, and a yellowish-white slough at the base of the wound. According to the patient’s medical history, he was taking broad-spectrum antibiotics and using a povidone–iodine dressing and local steroidal ointment following wound debridement. The patient worked as a laborer. The patient remembers walking barefoot or in improper footwear, but there was no history of trauma, bug bites, or punctures, and they had chewed tobacco for eighteen years. Over time, the region swelled, developed a slough, and began to leak foul-smelling fluid. Initially, there was no pain, but as the wound deepened and became infected, later discomfort was noticed. He chose selective treatment for a similar problem without careful intervention; he has been on medicine since the injury and has not made any progress. MTG was applied following a thorough assessment of the lesion and the patient’s agreement. Blood sugar levels ranged from 150 mg/dL to 180 mg/dL when fasting to 300 mg/dL after eating. Patients were instructed to apply the appropriate dressing after applying MTG to the wound twice a day. Additionally, the modern doctor recommended that diet and diabetes medication be continued at the same dosage. The wound was measured using a measuring scale on the 0th, 15th, and 30th day and photographed at a time point to compare it with the initial state. Furthermore, a pus sample was sent for culture and sensitivity (C/S) testing on the 0th and 30th day to determine the growth and evaluate the suppression of bacteria in the wound following treatment, i.e., to analyze the gel’s extra antibacterial action.

#### 3.3.3. Case 3

This 52-year-old female visited the same hospital on 1 May 2025, with MRD number 72722241. She had a single map-like, elongated wound on the dorsolateral aspect of her right foot for ten months, measuring roughly 7 cm in length, 3 cm in width, and 0.2 cm in depth (Figure 10C). The wound had a moderate yellowish exudate. She was receiving sporadic follow-up care after being diagnosed with type 2 diabetes mellitus nine years ago. The patient’s blood pressure was 106/68 mm Hg, temperature was 98 °F, and pulse rate was 78 beats per minute. Glycated hemoglobin (HbAlc 6.9%), Complete blood count, Renal Function Test, Liver Function Test, and lipid profiles were within normal ranges. When the wound was examined locally, it showed a single wound with an unclear edge, granulation tissue covering the wound base, some blackish discoloration along the wound edge, and hyperpigmented skin with early indications of peri-wound maceration. According to the patient’s medical history, he was taking broad-spectrum antibiotics and using a povidone–iodine dressing and local steroidal ointment following wound debridement. The patient worked as a factory laborer. The patient initially reported redness and mild swelling in the area, which was followed by the formation of a small blister that burst on its own. The wound was either ignored or went unnoticed in its early stages, but as time went on, it got larger and showed surrounding skin discoloration, an unpleasant-smelling discharge, and an infection. The patient denies having ever experienced trauma. She chose elective treatment for a similar problem without careful intervention; she has been on medicine since the injury and has not made any progress. and then visited the Sir Sunder Lal Hospital’s surgical outpatient department. MTG was applied following a thorough assessment of the wound and the patient’s consent. Blood sugar levels ranged from 140 to 170 mg/dL during fasting and up to 260 mg/dL after eating. Patients were instructed to apply the appropriate dressing after applying MTG to the wound twice a day. Additionally, the modern doctor recommended that diet and diabetes medication be continued at the same dosage. The wound was measured using a measuring scale on the 0th, 15th, and 30th day and photographed at a time point to compare it with the initial state. Furthermore, a pus sample was sent for culture and sensitivity (C/S) testing on the 0th and 30th day to determine the growth and evaluate the suppression of bacteria in the wound following treatment, i.e., to analyze the gel’s extra antibacterial action.

#### 3.3.4. Case 4

On 3 June 2025, a 48-year-old female patient with MRD number 7166140 had a single, elongated wound over the posteroinferior foot region that was about 7 cm long, 3 cm wide, and 0.2 cm deep. The wound extended to the plantar aspect of the right foot and had been there for a year (Figure 10D). Fifteen years ago, she had a diagnosis of type 2 diabetes mellitus and was receiving irregular follow-up care. The patient’s general examination showed that she was in good health, with a temperature of 98 °F, a pulse of 98 beats per minute, and a blood pressure of 150/90 mm Hg. Glycated hemoglobin (HbAlc 7.0%), Complete blood count, Renal Function Test, Liver Function Test, and lipid profiles were within normal ranges. At baseline, a single elongated wound was found over the posteroinferior foot region, extending to the plantar aspect of the right foot. The wound’s edges were ill-defined and rough, with mild yellowish exudation. The wound bed had a mix of granulation tissue and yellow slough. The surrounding skin was hyperpigmented, thickened, and exhibited early peri-wound maceration, indicating long-term tissue involvement. The patient’s treatment history shows that she was taking broad-spectrum antibiotics and using local steroidal ointment and a povidone–iodine bandage following wound debridement. The patient was a factory worker who was barefoot on the work site. Initially, the patient noticed redness and mild swelling in the area, followed by the development of a small boil that went unnoticed or was ignored in the early stage. Over time, the wound increased in size, with surrounding skin discoloration and foul-smelling discharge, and signs of infection. The patient denies any history of trauma. She has been on medicine since the injury and has made no progress; she chose elective treatment for a similar issue without caution. Then, he visited Sir Sunder Lal Hospital’s surgical outpatient department. After receiving the patient’s consent and doing a thorough examination of the wound, MTG was applied. Blood sugar levels ranged from 140 mg/dL to 170 mg/dL during fasting and post-prandial up to 269 mg/dL. Patients were instructed to apply proper dressing and ointment over the wound twice a day, followed by a proper dressing. In addition, oral diabetes medication and diet should be continued at the same dose as recommended by a modern physician. The wound was measured using a measuring scale on the 0th, 15th, and 30th day and photographed at a time point to compare it with the initial state. Furthermore, a pus sample was sent for culture and sensitivity (C/S) testing on the 0th and 30th day to determine the growth and evaluate the suppression of bacteria in the wound following treatment, i.e., to analyze the gel’s extra antibacterial action.

#### 3.3.5. Case 5

This 54-year-old male patient presented to our care on 3 June 2025. The patient was visited at the hospital, MRD number was 7329479, with a single elongated wound of approximately 5 cm in length, 3 cm in width, and 0.5 cm in depth across the plantar region of the right foot for 8 months (Figure 10E). He was diagnosed with type 2 diabetes mellitus 11 years ago and has obtained irregular follow-up treatment. A general examination of the patient revealed that she was in good health, with a pulse rate of 90/min, a temperature of 98 °F, and a blood pressure of 142/74 mm Hg. Other biochemical markers, including glycated hemoglobin (HbAlc 8.4%), Complete blood count, Renal Function Test, Liver Function Test, and lipid profiles, were within normal ranges. Local inspection revealed a single oval, elongated wound over the plantar portion of the foot, with ill-defined and irregular edges. The wound bed displayed a mixed tissue pattern, with healthy red granulation tissue and yellowish slough adhering to the base. The wound showed full-thickness tissue loss, with subcutaneous tissue involvement, culminating in a moderate depth. There was no obvious evidence of tendon, muscle, or bone. The patient’s treatment history shows that he was taking broad-spectrum antibiotics and using local steroidal ointment and a povidone–iodine bandage following wound debridement. The patient was working as a laborer in a coal mine, where he spent extended periods of time walking or standing. Initially, the patient noticed skin breakdown and mild discharge in the area, followed by the development of a small wound that went unnoticed or was ignored in the early stage. Over time the wound increased in size, with surrounding skin discoloration and foul-smelling discharge, and signs of infection. The patient denies any history of trauma. He had been on medicine since the injury and had made no progress, so he chose treatment for a similar issue rather than cautious intervention. Then, she came to Sir Sunder Lal Hospital’s surgical outpatient department. After taking the patient’s consent and performing a thorough assessment of the wound, MTG was applied. The patient’s blood sugar level ranged from 150 to 170 mg/dL in the fasting state and post-prandial up to 200 mg/dL. The patient was instructed to apply the MTG around the wound twice daily followed by an appropriate dressing. In addition, the patient was advised to continue oral medication for diabetes mellitus and maintain the prescribed diet as recommended by the modern physicians without any change in doses. The wound size was measured using a measuring scale on the 0, 15th and 30th and photographs were taken at each time point to compare it with the initial state. Furthermore, a pus sample was sent for culture and sensitivity (C/S) testing on the 0th and 30th day to determine the growth and evaluate the suppression of bacteria in the wound following treatment, i.e., to analyze the gel’s extra antibacterial action. All clinical cases exhibited a progressive and measurable reduction in wound dimensions throughout the 30th day treatment period. Significant wound contraction was evident by day 15, with complete epithelialization achieved in three of the five cases by day 30. Baseline microbiological assessment confirmed the presence of pathogenic bacterial colonization in all wounds. At the end of the treatment period, complete bacterial clearance was observed in two cases, as shown in Table 4, while the remaining cases demonstrated either an altered microbial profile or reduced persistence of pathogenic organisms, concomitant with substantial wound healing. Collectively, these findings indicate that the intervention effectively promoted wound healing while exerting a notable antibacterial effect.

### 3.4. Statistical Analysis

The mean ± SD (*n* = 3) was used to display the experiment data. The statistical computation was done using GraphPad Prism 5.0. The statistical significance between the groups was examined using a one-way ANOVA. The following statistically significant levels were considered as non-significant (ns) (*p* ≥ 0.05) and significant: * (*p* < 0.05), ** (*p* < 0.01), and *** (*p* < 0.001).

## 4. Discussion

Diabetic wound healing is a multicellular process including homeostasis, inflammation, cell proliferation, and restoration [65,66]. Non-healing diabetic wounds affect approximately 25% of all diabetes patients, which frequently leads to limb amputation [67,68]. Herein, we have developed a Medicated Topical Gel for diabetic wound healing. Due to rising antibiotic resistance, many currently available drugs have minimal impact on diabetic wound healing [69]. However, the MTG has incorporated two ayurvedic herbs, *D. hamiltonii* root powder and *B. aegyptiaca* seed oil. Herbal products and their phytoconstituents enhance the growth factors which promote cell proliferation, migration, angiogenesis [70], and wound contractions [71,72]. MTG is easily dispersed, effective, and stable for a year at room temperature. Its morphology was uniformly distributed, and its crystalline peaks were determined to be thermally stable. Previous study on dermal toxicity and allergic responses explained the histological features [73], but upon dermal acute application of DHRE, BASO, and MTG at up to 2000 mg/kg for 14 days, they do not show any transient or notable toxicity findings. In repeated dosing, R-DHRE, R-BASO, and R-MTG show minute changes compared to control. These observational changes with repeated dosing are common in the dermal and subcutaneous area upon long-term exposure to the drug [74]. Within the experimental duration, no advanced toxic characteristic was observed. Diabetic wound healing is closely linked with hypoxia, neovascularization, impaired cell migration, infections, neuropathy, poor vascular network, and hyperglycemia [75,76,77]. Diabetic wounds also demonstrate increased expression of pro-inflammatory cytokines, including TNF-α and IL-6, which sustain chronic inflammation and damage the peripheral nerves and reduce the blood supply to the lower extremities [78]. The persistence of inflammation and bacterial load further complicates the healing. It may cause difficulties in controlling the chronic inflammation in a diabetic wound. Sub-infective levels of bacteria may support normal wound healing by enhancing neutrophil and macrophage infiltration and promoting collagen synthesis [79]. Hyperglycemia plays a major role in the induced production of reactive oxygen species [80] and affects many pathways, such as protein kinase, hexosamine, and glycation production [81]. This study reveals that the enhanced expression of VEGFR-2 suggests that the MTG stimulates angiogenesis more effectively than the standard treatment. MTG stimulates regeneration, triggering robust VEGF/VEGFR-2 signaling as new vessels form or as damaged tissue attempts to recover. The significant enhancement of VEGFR-2 expression in the MTG group highlights its ability to activate pro-angiogenic signaling, likely through stimulation of the VEGF/VEGFR-2 pathway, which promotes endothelial migration and capillary sprouting essential for wound closure [82]. Inflammation is another critical event in the wound healing process, but prolonged inflammation can delay tissue repair [83]. In contrast, the BASO and DHRE groups displayed only moderate reductions in cytokine expression, indicating a lesser degree of control over inflammation. Inflammations indicators such as TNF-α and IL-6 have demonstrated resistance to insulin; IL-6 is a trigger to several glucocorticoid receptors [84]. The concurrent upregulation of VEGFR-2 and downregulation of TNF-α and IL-6 in the MTG-treated group highlights its dual action, enhancing angiogenesis while mitigating inflammation [85]. Indicators of inflammation, such as TNF-α and IL-6, have been reported to increase in obese rodents and in those demonstrating resistance to insulin. IL-6 is believed to trigger several glucocorticoid receptors, applied on a paracrine influence on the response of adipose tissue to insulin and to promote serum glucagon levels [86]. The MTG group exhibited significant upregulation of VEGFR-2 and marked downregulation of TNF-α and IL-6 compared to the negative control and positive control, indicating enhanced angiogenesis and reduced inflammation during the wound healing process [87]. During the wound healing process, edema, epidermal differentiation, inflammation, collagen maturity, granulation, and vascularization are involved [88,89,90]. Preclinical results demonstrated that MTG achieves superior healing efficacy, with a wound closure rate by Day 24. MTG may target multiple mechanisms involved in wound healing, including inhibition of matrix metalloproteinases (MMPs), stimulation of collagen production, reduction in oxidative stress and inflammatory responses (by decreasing IL-6 and TNF-α levels), antimicrobial activity, promotion of neovascularization (through increased VEGFR-2 expression), and stimulation of hair follicle development [91]. These findings were further supported by histological evidence, which showed significantly lower wound scores compared to the negative control group, indicating enhanced healing. Clinical observations from all five cases demonstrated a reduction in wound size, increased granulation tissue formation, improved re-epithelialization, and decreased bacterial load. Culture and sensitivity (C/S) testing revealed that *Staphylococcus aureus* was the most commonly detected bacterium at the wound site during the initial assessment; however, it was completely absent after treatment throughout the 30-day study period. Overall, these results indicate that the Medicated Topical Gel (MTG) could represent a promising evidence-based therapeutic option for the treatment of chronic diabetic wounds.

## 5. Conclusions

This research emphasizes the increasing complexity of diabetic wounds in the current clinical scenario and highlights the therapeutic potential of the developed MTG. The MTG demonstrated significant efficacy in the management of chronic diabetic wounds as well as multidrug-resistant (MDR) bacterial-infected wounds, without showing any adverse effects when compared to marketed formulations. The developed gel exhibited a sustained release of the therapeutic agents at the wound site, resulting in accelerated wound healing within 24 days. Molecular analysis by Western blotting revealed upregulation of key angiogenic markers, particularly VEGFR-2, along with significant downregulation of pro-inflammatory cytokines IL-6 and TNF-α in MTG-treated groups, indicating effective modulation of inflammation and enhanced angiogenesis. Furthermore, in the clinical case series conducted over 30 days, MTG application resulted in a marked reduction in pus formation and effective elimination of several MDR bacterial strains, confirming its antibacterial activity. Overall, the MTG-based wound dressing demonstrates promising antibacterial and anti-inflammatory properties with substantial wound healing potential. This formulation may serve as a foundation for the development of future advanced therapeutic strategies for chronic diabetic and infected wounds.

## 6. Patent

Title of the Indian patent: Topical composition and method for skin disorders, Application No: 202411101832, Published Date: 28 March 2025.

## Figures and Tables

**Table 1:** The composition of the MTG has been presented.

S. No.	Groups	Medicated Oil	Beewax	SEPINEO^TM^ P 600
1	Blank gel	-	3.4%	1%
2	MTG	15.60%	3.40%	1%

**Table 2:** ATR-FTIR of the MTG (Medicated Topical Gel).

Band Position (cm^−2^)	Vibration Mode	Functional Groups
3787	O-H Stretching	Hydroxyl Group
3454	O-H Stretching	
2923	C-H Stretching	Aliphatic methylene group
1614	C=C Stretching	Aromatic ring
1404	O-H Bending	Alcoholic groups
1242	C-N Stretching	Amide/Alkaloid groups
1078	C-O Stretching	Ether groups
1059	C-O Stretching	
778	C=O Bending	Amide groups
616	C=O Bending	
522	C-O-C	Ether groups

**Table 3:** Comparative biochemical parameters of acute (**A**) and repeated (**B**) dosing.

(A)				
Biochemical Parameter	Control	DHRE	BASO	MTG
Alkaline phosphates (IU/Lt.)	315	381	262	335
Albumin (gm/dL)	3.9	3.6	3.8	3.7
Globulin (gm/dL)	3.7	3.2	3.2	3.6
AST (IU/Lt.)	145	156	126	176
ALT (IU/Lt.)	75	66	61	71
Total bilirubin (mg/dL)	0.2	0.4	0.4	0.2
Blood urea (mg/dL)	39	40.7	31.8	31.5
Creatinine (mg/dL)	0.74	0.7	0.73	0.71
Sodium (mEq/Lt.)	143	143	149	143
Potassium (mEq/Lt.)	6.5	6.2	6.3	7.1
Chloride (mEq/Lt.)	104	106	105	104
Calcium (mg/dL)	10.1	10.4	10.7	10.6
Phosphorus (mg/dL)	6.6	7.3	7.3	6.9
(**B**)				
**Biochemical Parameter**	**R-Control**	**R-DHRE**	**R-BASO**	**R-MTG**
Alkaline phosphates (IU/Lt.)	429	399	354	302
Albumin (gm/dL)	4.6	4.9	4.8	4.7
Globulin (gm/dL)	2.5	2.3	2.5	2.3
AST (IU/Lt.)	116	112	127	124
ALT (IU/Lt.)	44	48	56	41
Total bilirubin (mg/dL)	0.2	0.2	0.3	0.3
Blood urea (mg/dL)	40.6	43.4	40.1	46
Creatinine (mg/dL)	0.76	0.78	0.66	0.78
Sodium (mEq/Lt.)	144	145	144	140
Potassium (mEq/Lt.)	4.2	4	4.1	4.2
Chloride (mEq/Lt.)	104	105	104	103
Calcium (mg/dL)	9.9	11	9.8	9.9
Phosphorus (mg/dL)	5.6	5.3	4.3	6.4

**Table 4:** Wound size measurements and bacterial growth at different time points.

Cases	0 Day Wound Size (cm^3^)	15th Day Wound Size (cm^3^)	30th Day Wound Size (cm^3^)	Site of the Wound	Bacterial Growth	
					At 0 Day	30th Day
Case 1 (MRD No. 6877209)	2 × 1.4 × 0.5	1.5 × 1.0 × 0.2	1 × 0.5 × 0.1	Plantar surface of the right great toe	*Klebsiella pneumoniae* and *Staphylococcus aureus*	*Escherichia coli*
Case 2 (MRD No. 6994028)	2.2 × 1.8 × 2	2.2 × 1.6 × 0.6	1.5 × 1.0 × 0.1	Central plantar surface of the right heel	*Citrobacter freundii*	No growth
Case 3 (MRD No. 7272241)	7 × 3 × 0.2	1 × 0.5 × 0.1	Healed	Dorsolateral aspect of the right foot	*Staphylococcus aureus* and *Streptococcus pyogenes*	*Escherichia coli*
Case 4 (MRD No. 7166140)	8 × 3 × 0.5	5 × 1 × 0.2	Healed	Posterior-inferior heel region extending towards the plantar aspect of the right foot	*Pseudomonas aeruginosa* and *Staphylococcus aureus*	No growth
Case 5 (MRD No. 7329479)	5 × 3 × 0.5	4 × 1 × 0.1	Healed	Plantar region of the right foot	*Staphylococcus aureus* and *Pseudomonas aeruginosa*	*Staphylococcus aureus*

## Data Availability

The datasets generated and/or analyzed are available from the corresponding author upon request.

## Supplementary Materials

The following supporting information can be downloaded at: https://www.mdpi.com/article/10.3390/pharmaceutics18040429/s1, Figure S1: Represent HPLC chromatogram, of biomarkers (a) Caffeic acid, (b) Diosgenin, and (c) Linoleic acid.

## Abbreviations

T2DM	Type 2 Diabetic mellitus
STZ	Streptozotocin
GC-MS	Gas Chromatography Mass Spectrometry
MTG	Medicated Topical Gel
DHRE	Decalepis hamiltonii Root Extract
BASO	*Balanites aegyptiaca* Seed Oil
R-DHRE	Repeated *Decalepis hamiltonii* Root Extract
R-BASO	Repeated *Balanites aegyptiaca* Seed Oil
FE-SEM	Field Emission Scanning Electron Microscope
DSC	Differential Scanning Calorimetry
TGA	Thermogravimetry Analysis
FTIR	Fourier Transform Infrared Spectrometry
HPLC	High Performance Liquid Chromatography
CTRI	Clinical Trial Registry India
CCSEA	Committee for Control and Supervision of Experiments on Animals
VEGFR-2	Vascular Endothelial Growth Factor Receptor 2
TNF-α	Tumor Necrosis Factor
IL-6	Interleukin-6
PXRD	Powder X-Ray Diffraction
