# Supplementary material for: Evidence-Based Translational Strategy of Medicated Topical Gel for Diabetic Wound Management
Source: Pharmaceutics. 2026 Mar 31;18(4):429. doi: 10.3390/pharmaceutics18040429 (PMC13118908; doi:10.3390/pharmaceutics18040429)
Supplement: Supplementary file 1 [file pharmaceutics-18-00429-s001.zip › pharmaceutics-4182638-supplementary.pdf]

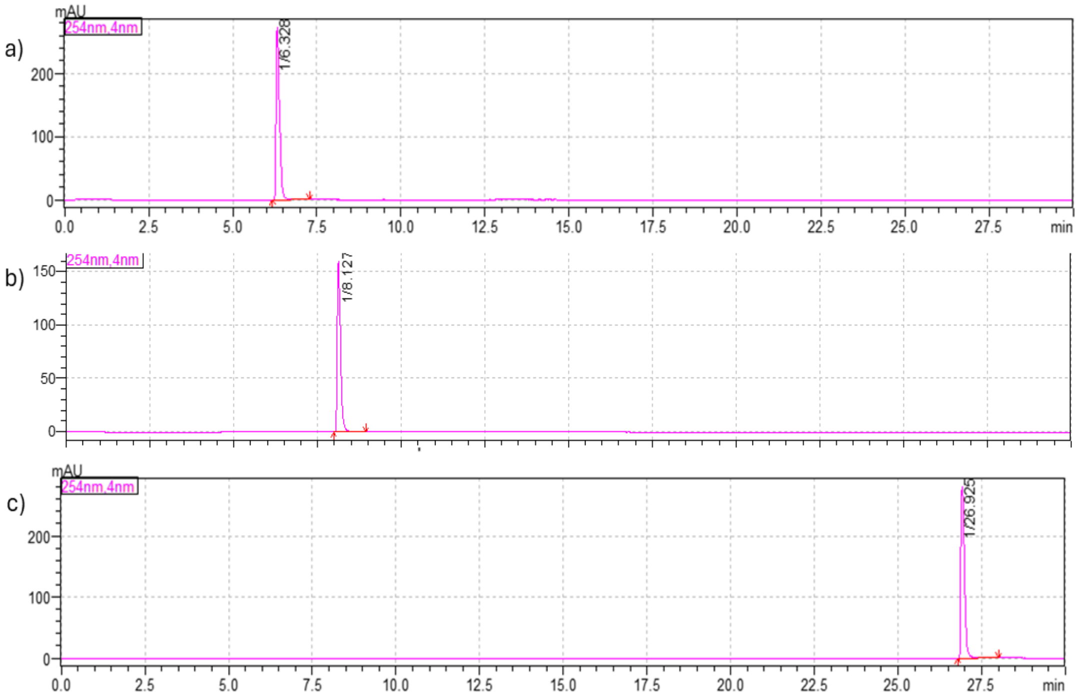

Figure S1: Represent HPLC chromatogram, of biomarkers a) Caffeic acid, b) Diosgenin, and c) Linoleic acid
